# Supplementary material for: Genome-scale metabolic reconstruction and metabolic versatility of an obligate methanotroph Methylococcus capsulatus str. Bath
Source: PeerJ. 2019 Jun 14;7:e6685. doi: 10.7717/peerj.6685 (PMC6613435; doi:10.7717/peerj.6685)
Supplement: Supplemental Information 3 [file peerj-07-6685-s003.pdf]

Supplementary Table 2: List of reactions included in the GSMM without a GPR

Reaction ID	Subsystem	Comment
R08159	Fatty acid Metabolism	Gap Filling
R03370	Fatty acid Metabolism	Gap Filling
R02814	Fatty acid Metabolism	Gap Filling
R00258	Arginine Biosynthesis	Gap Filling
R00485	Alanine, asp, glutamate synthesis	Gap Filling
R00576	Alanine, asp, glutamate synthesis	Gap Filling
R01215	Valine, leucine, isoleucine	Gap Filling
R00345	Pyruvate Metaboilsm	Gap Filling
R08716	Porphyrin and chlorophyll metabolism	Gap Filling
R08676	ADP glucose	Gap Filling
R07410	Nicotinate and nicotinamide metabolism	Gap Filling
R07407	Nicotinate and nicotinamide metabolism	Gap Filling
R07280	Riboflavin metabolism	Gap Filling
R05705	Riboflavin metabolism	Gap Filling
R05639	Steroid biosynthesis	Gap Filling
R03199	Steroid biosynthesis	Gap Filling
R03013	Histidine Metabolism	Gap Filling
R02999	Porphyrin and chlorophyll metabolism	Gap Filling
R02874	Steroid biosynthesis	Gap Filling
R01771	Glycine, serine and threonin	Gap Filling
R01706	Fatty Acid Metabolism	Gap Filling
R01655	One carbon Pool	Gap Filling
R01398	Arginine Metabolism	Gap Filling
R00941	One carbon Pool	Gap Filling
R00847	Glycerolipid metabolism	Gap Filling
R00806	Starch and Sucrose metabolism	Gap Filling
R00528	Sulfer metabolism	Gap Filling
R00149	Arginine Metabolism	Gap Filling
R_formylTHMPT_formate	THMPT cycle	Gap Filling
Biomass Reaction	-	Biomass Synthesis
Rspont2	Arginine and proline metabolism	Spontaneous Reaction(Kegg db)
Starch_synthesis	Starch Synthesis and Degradation	Starch synthesis
Starch_degredation	Starch Synthesis and Degradation	Starch degradation
RC08362	Fatty Acid Synthesis	Reported (Makula 1978)
RCL160161	Fatty Acid Synthesis	
RCL161140	Fatty Acid Synthesis	
RCL161161	Fatty Acid Synthesis	
RPC160161	Fatty Acid Synthesis	
RPC161140	Fatty Acid Synthesis	
RPC161161	Fatty Acid Synthesis	
RPE160161	Fatty Acid Synthesis	
RPE161140	Fatty Acid Synthesis	
RPE161161	Fatty Acid Synthesis	
RPG160161	Fatty Acid Synthesis	
RPG161140	Fatty Acid Synthesis	
RPG161161	Fatty Acid Synthesis	

Makula, R A. 1978. “Phospholipid Composition of Methane-Utilizing Bacteria.” *Journal of bacteriology* 134(3): 771–77. http://www.ncbi.nlm.nih.gov/pubmed/96101 (April 14, 2018).
