# Supplementary figures and images for: Genome-scale metabolic reconstruction and metabolic versatility of an obligate methanotroph Methylococcus capsulatus str. Bath
Source: PeerJ. 2019 Jun 14;7:e6685. doi: 10.7717/peerj.6685 (PMC6613435; doi:10.7717/peerj.6685)

**Supplementary Figure S1.** Top 35 highly connected metabolites in the *i*MC535 model.

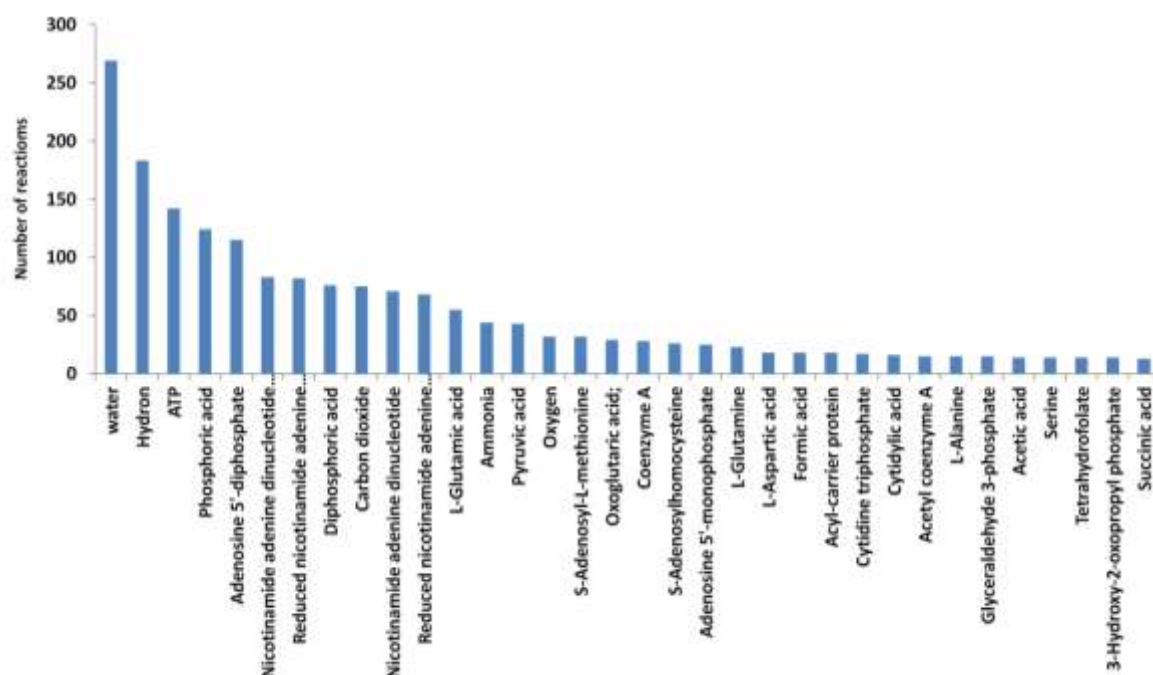

Supplement: Supplemental Information 8 [file peerj-07-6685-s008.pdf]
