# Supplementary material for: Genome-scale metabolic reconstruction and metabolic versatility of an obligate methanotroph Methylococcus capsulatus str. Bath
Source: PeerJ. 2019 Jun 14;7:e6685. doi: 10.7717/peerj.6685 (PMC6613435; doi:10.7717/peerj.6685)

Supplementary Figure S2. Distribution of all the reactions of both models along various subsystems

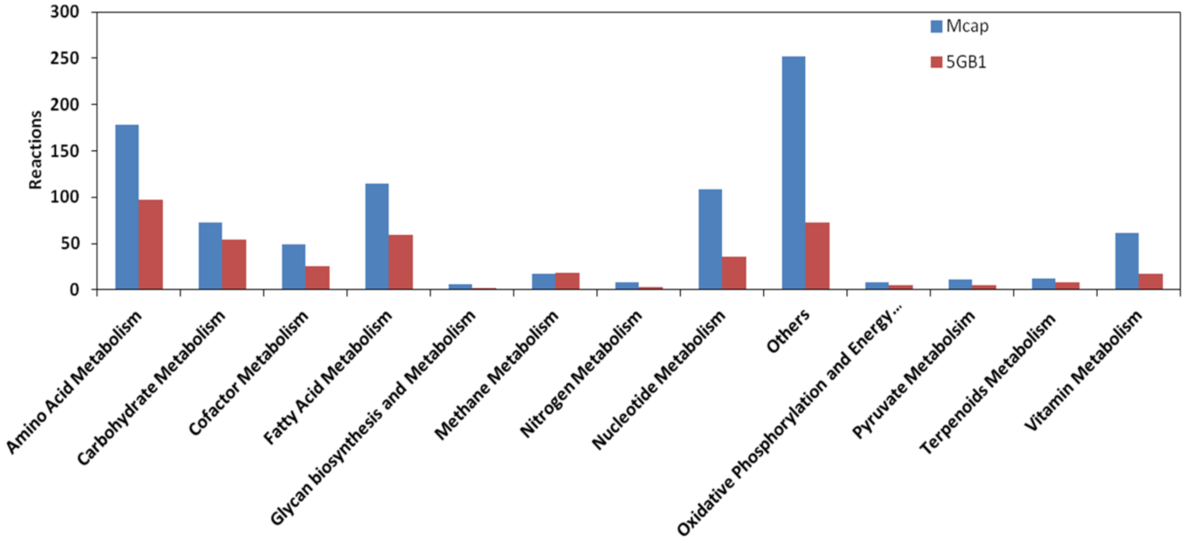

Supplement: Supplemental Information 9 [file peerj-07-6685-s009.pdf]
