# Supplementary material for: Genome-scale metabolic reconstruction and metabolic versatility of an obligate methanotroph Methylococcus capsulatus str. Bath
Source: PeerJ. 2019 Jun 14;7:e6685. doi: 10.7717/peerj.6685 (PMC6613435; doi:10.7717/peerj.6685)
Supplement: Supplemental Information 10 [file peerj-07-6685-s010.docx]

# ***Flux balance analysis***

model = xls2model('IMC535.xls');

model = changeObjective(model,'Biomass_reaction');

model = changeRxnBounds(model, {'CH4_tx'}, -18.5, 'b');

fba = optimizeCbModel(model,'max')

# ***Alternate Carbon Source***

model = xls2model('IMC535.xls');

model = changeRxnBounds(model, {'CH4_tx'}, 0, 'b');

model = changeRxnBounds(model, {'Pyr_tx'}, -18.5, ‘l’);

model = changeRxnBounds(model, {'Lact_tx'}, -18.5, ‘l’);

model = changeRxnBounds(model, {'Formate_tx'}, -18.5, ‘l’);

model = changeRxnBounds(model, {'EtOH_tx'}, -18.5, ‘l’);

model = changeRxnBounds(model, {'CO2_tx'}, -18.5, 'l');

model = changeRxnBounds(model, {'Ac_tx'}, -18.5, ‘l’);

model = changeRxnBounds(model, {'Suc_tx'}, -18.5, ‘l’);

model = changeRxnBounds(model, {'Ac_tx'}, -18.5, 'l');

model = changeObjective(model,'Biomass_reaction');

fba = optimizeCbModel(model,'max')

printFluxVector(model,fba.x)

#***Alternate Nitrogen Source***

model = xls2model('IMC535.xls');

model = changeRxnBounds(model, {'CH4_tx'}, -18.5, 'b');

model = changeRxnBounds(model, {'nitrite_tx'}, -0, ‘l’);

model = changeRxnBounds(model, {'nitrate_tx'}, -0, ‘l’);

model = changeRxnBounds(model, {'NH3_tx'}, -0, ‘l’);

model = changeRxnBounds(model, {'N2_tx'}, -1, ‘l’);

model = changeObjective(model,'Biomass_reaction');

fba = optimizeCbModel(model,'max')

***#Growth on amino acids***

model = changeRxnBounds(model, {'Valine_tx'}, -10, 'l'); valine

model = changeRxnBounds(model, {'alanine_tx'}, -10, 'l'); Alanine -> 0.427 -> -3.93

model = changeRxnBounds(model, {'Aspartate_tx'}, -10, 'l'); Aspartate -> 0.427 -> -3.93

model = changeRxnBounds(model, {'Aspargine_tx'}, -10, 'l'); Asparagine -> 0.308 -> -1.42

model = changeRxnBounds(model, {'Cysteine_tx'}, -10, 'l'); Cysteine -> 0.5770 -> -5.31

model = changeRxnBounds(model, {'Glutamate_tx'}, -10, 'l'); Glutamate -> 0.698 -> -6.425

model = changeRxnBounds(model, {'Glutamine_tx'}, -10, 'l'); Glutamine -> 0.36 -> -1.658

model = changeRxnBounds(model, {'Urea_tx'}, -10, 'l'); no growth

model = changeRxnBounds(model, {'R1'}, 0, 'b'); valine

model = changeRxnBounds(model, {'R2'}, 0, 'b'); Alanine -> 0.427 -> -3.93

model = changeRxnBounds(model, {'R3'}, 0, 'b'); Aspartate -> 0.427 -> -3.93

model = changeRxnBounds(model, {'R4'}, 0, 'b'); Asparagine -> 0.308 -> -1.42

model = changeRxnBounds(model, {'R5'}, 0, 'b'); Cysteine -> 0.5770 -> -5.31

model = changeRxnBounds(model, {'R6'}, 0, 'b'); Glutamate -> 0.698 -> -6.425

model = changeRxnBounds(model, {'R7'}, 0, 'b'); Glutamine -> 0.36 -> -1.658

fba = optimizeCbModel(model,'max')

[minFlux,maxFlux] = fluxVariability(model,100);

printFluxVector(model,fba.x)

# ***Sulfur source***

model = xls2model('IMC535.xls');

model = changeRxnBounds(model, {'CH4_tx'}, -0, 'b');

model = changeRxnBounds(model, {'sulfate_tx'}, -0, 'b');

model = changeRxnBounds(model, {'R_H2S'}, -0, 'b');

model = changeObjective(model,'Biomass_reaction');

fba = optimizeCbModel(model,'max')

# ***Double gene deletion analysis***

model = xls2model('iMC535.xls');

model = changeObjective(model,'Biomass_reaction');

model = changeRxnBounds(model, {'CH4_tx'}, -18.5, 'b');

fba = optimizeCbModel(model,'max')

[grRatiodouble, grRateKOdouble, grRateWTdouble] = doubleGeneDeletion(model);

interactions = findEpistaticInteractions(model,grRatiodouble);

nInteractions = sum(interactions);

plot(sort(nInteractions(nInteractions > 0)),'-');

xlabel(' Gene')

ylabel('number of synthetic lethal or synthetic sick interactions')

***# Gene Deletion analysis***

model = xls2model('iMC535.xls');

model = changeObjective(model,'Biomass_reaction');

model = changeRxnBounds(model, {'CH4_tx'}, -18.5, 'b');

fba = optimizeCbModel(model,'max')

***# Gene deletion analysis in EMP - 0 condition***

model = xls2model(‘iMC535.xls');

model = changeObjective(model,'Biomass_reaction');

model = changeRxnBounds(model, {'CH4_tx'}, -18.5, 'b');

ReacsEMP = {'R02740','R04779','R01070','R02739','R05805','R00756'};

model = changeRxnBounds(model, ReacsEMP, 0, 'b');

fba = optimizeCbModel(model,'max')

# ***Gene deletion analysis in N source condition***

model = xls2model(‘iMC535.xls');

model = changeRxnBounds(model, {'CH4_tx'}, -18.5, 'b');

model = changeRxnBounds(model, {'nitrite_tx'}, -0, 'b'); grows -1.82 max.

model = changeRxnBounds(model, {'nitrate_tx'}, -0, 'b'); grows -1.82 max.

model = changeRxnBounds(model, {'NH3_tx'}, 000, 'b'); grows -2.2 max.

model = changeObjective(model,'Biomass_reaction');

fba = optimizeCbModel(model,'max')

# ***Codes for Gene Deletion Studies***

[grRatio,grRateKO,grRateWT,hasEffect,delRxns,fluxSolution] = singleGeneDeletion(model);

grRatioMOMA= singleGeneDeletion(model,'lMOMA');

plot sorted (mutant growth)/(wild type growth)

plot(1:length(grRatio), [sort(grRatio)],'.');

xlabel(' Gene')

ylabel('growth rate of mutant relative to wild type')

figure

plot(1:length(grRatioMOMA), [sort(grRatioMOMA)],'.');

xlabel(' Gene')

ylabel('growth rate of mutant relative to wild type (MOMA)')

# ***Codes* for Detecting effect of gene deletion on model structure.**

hasEffectGene = {};

has_NoEffectGene = {};

j = 0;

k = 0;

for i = 1:length(model.genes)

if hasEffect(i) == 1

j = j+1;

hasEffectGene(j,1) = model.genes(i);

else

k = k+1;

has_NoEffectGene(k,1) = model.genes(i);

end

end

***# list of essential genes and Non essential Genes***

EssentialGenes = {};

NonEssentialGenes = {};

j = 0;

k = 0;

for i = 1:length(grRateKO)

if grRateKO(i) < 1e-6 | isnan(grRateKO(i))

j = j + 1;

EssentialGenes(j,1) = model.genes(i);

else

k = k + 1;

NonEssentialGenes(k,1) = model.genes(i);

end

end

***# THMPT Pathway***

model = xls2model('IMC535.xls');

model = changeObjective(model,'Biomass_reaction');

ReacsTHMPT = {'R08058','R08059','R03464','R03390'};

model = changeRxnBounds(model, ReacsTHMPT, 0, 'b');

model = changeRxnBounds(model, {'CH4_tx'}, -18.5, 'b');

model = changeRxnBounds(model, {'R00943'}, 0, 'b');

model = changeRxnBounds(model, {'R04325'}, 0, 'b');

model = changeRxnBounds(model, {'R04560'}, 0, 'b');

fba = optimizeCbModel(model,'max')

[minFlux,maxFlux] = fluxVariability(model,100);

***# Flux correlation analysis***

model = xls2model(‘iMC535.xls');

model = changeObjective(model,'Biomass_reaction');

model = changeRxnBounds(model, {'CH4_tx'}, -18.5, 'b');

fba = optimizeCbModel(model,'max')

# ***To identify correlated reactions***

growthRate = fba.f;

[modelSampling,samples] = sampleCbModel(model,’iMC535.xls_flux');

[sets,setNumber] = identifyCorrelSets(modelSampling,samples);

selectRxns = setNumber > 0 & setNumber <=10;

outputRxnList = model.rxns(selectRxns);

outputSetNumber = setNumber(selectRxns);

outputNetworkCytoscape(model, 'iND750_correlSets');
